# Supplementary material for: Content-rich biological network constructed by mining PubMed abstracts
Source: BMC Bioinformatics. 2004 Oct 8;5:147. doi: 10.1186/1471-2105-5-147 (PMC528731; doi:10.1186/1471-2105-5-147)
Supplement: Additional File 2 — The original results of the above study (non-essential files are deleted to keep the file size under the limit set by BMC bioinformatics). [file 1471-2105-5-147-S2.bz2 › chilibotAdditionalFile2/dip30/618ID8736558E11002/html/TXN_APEX1.html]

 


 **TXN** and **APEX1** 
  
Found 454 abstracts in PubMed, retrieved 30.  
 

 What does Google say? 
 PDF only 
| .edu only 

---

**Interactive relationship** (e.g. stimulation, inhibition, etc)

**Neutral relationship**- T7 DNA  [ **APEX1** ]  polymerase Real time kinetics of the interaction between its two subunits, escherichia coli thioredoxin  [ **TXN** ]  and gene 5 protein of phage T7.  Ref: 12670954 J Biol Chem< MedlineTA>J Biol Chem,
- A fusion protein Trx  [ **TXN** ]  KasT clearly showed DNA  [ **APEX1** ]  binding activity with the intergenic region of kasU kasJ, suggesting that KasT is a pathway specific regulator of the KSM biosynthesis gene cluster.  Ref: 12617515 J Antibiot Tokyo , 2002
- The changes observed in spore DNA  [ **APEX1** ]  photochemistry at 1 2 Pa in vivo were not reproduced by irradiation of naked DNA  [ **APEX1** ]  or SspC DNA  [ **APEX1** ]  complexes in vitro, suggesting that factors other than SASP  [ **TXN** ]  are involved in spore DNA  [ **APEX1** ]  photochemistry at low pressure.  Ref: 12593780 Astrobiology< MedlineTA>Astrobiology, 2002
- Gene 5 protein of bacteriophage T7 is a non processive DNA  [ **APEX1** ]  polymerase which acquires high processivity by binding to Escherichia italic coli italic thioredoxin  [ **TXN** ] .  Ref: 12692131 J Biol Chem< MedlineTA>J Biol Chem, 2002
- However, gp5 S S trx  [ **TXN** ]  has only one third the polymerase activity of gp5 trx  [ **TXN** ]  on single stranded M13 DNA  [ **APEX1** ] .  Ref: 12692131 J Biol Chem< MedlineTA>J Biol Chem, 2002
- Gp5 S S trx  [ **TXN** ]  has difficulty polymerizing nucleotides through sites of secondary structure on M13 DNA  [ **APEX1** ]  and stalls at these sites, resulting in an apparently lower processivity.  Ref: 12692131 J Biol Chem< MedlineTA>J Biol Chem, 2002
- It is demonstrated that thioredoxin  [ **TXN** ]  2 of R. capsulatus can partially replace the thioredoxin  [ **TXN** ]  1 function as a hydrogen donor for methionine sulfoxide reductase but cannot replace thioredoxin  [ **TXN** ]  1 as a subunit of phage T7 DNA  [ **APEX1** ]  polymerase.  Ref: 12624204 Microbiology, 2003
- Myocardial TRX  [ **TXN** ]  was upregulated in myocarditis and cardiomyopathies with active necrotic stage associated with DNA  [ **APEX1** ]  damage, which may reflect the oxidative stress overload in hemodynamically uncontrolled status.  Ref: 12870673 Mol Cell Biochem, 2003
- However, gp5 S S trx  [ **TXN** ]  has an identical processivity and rate of elongation when E. coli single stranded DNA  [ **APEX1** ]  binding protein SSB protein is used to remove secondary structure from M13 DNA  [ **APEX1** ] .  Ref: 12692131 J Biol Chem< MedlineTA>J Biol Chem, 2003
- T7 phage DNA  [ **APEX1** ]  polymerase is a tight 1 1 complex of the gene 5 protein g5p 1 80 kDa of phage T7 and thioredoxin  [ **TXN** ]  12 kDa from the Escherichia coli host.  Ref: 12670954 J Biol Chem< MedlineTA>J Biol Chem, 2003
- DNA  [ **APEX1** ]  mismatch repair gene mutS 2.56 kb was PCR modified and cloned into a secretive prokaryotic expression vector pET32a which carries a N terminal His.tag and thioredoxin  [ **TXN** ]  sequence.  Ref: 12561194 Sheng Wu Gong Cheng Xue Bao, 2002
- Trx  [ **TXN** ]  1 transfected MCF 7 cells showed decreased Nrf 2 PMF 1 DNA  [ **APEX1** ]  binding without a change in Nrf 2 or PMF 1 protein expression.  Ref: 12804587 Biochem Biophys Res Commun, 2003
- Recombinant Bm TRX  [ **TXN** ]  1 had thiodisulfide reductase activity, as measured by the reduction of insulin, and protected DNA  [ **APEX1** ]  from the nicking activity of oxygen radicals.  Ref: 12819103 Infect Immun, 2003
- Therefore, increases in nuclear Trx  [ **TXN** ]  and Ref 1  [ **APEX1** ]  during aging may result in the up regulation of redox sensitive transcription factors, such as NF kappaB or AP 1, via the interaction of Ref 1  [ **APEX1** ]  and Trx  [ **TXN** ]  in a redox dependent manner.  Ref: 12742531 Exp Gerontol, 2003

**Non-interactive relationship** (e.g. studied together, co-existance, homology, etc.)

- In this study, wild type spores, mutant spores lacking alpha beta type small, acid soluble spore proteins SASP  [ **TXN** ] , naked DNA  [ **APEX1** ] , and complexes between SASP  [ **TXN** ]  SspC and DNA  [ **APEX1** ]  were exposed simultaneously to UV 254 nm at intermediate pressure 1 2 Pa, and the UV photoproducts cis, syn thymine thymine cyclobutane dimer c, sTT, trans, syn thymine thymine cyclobutane dimer t, sTT, and spore photoproduct SP were quantified.  Ref: 12593780 Astrobiology< MedlineTA>Astrobiology, 2003
- FACS analysis of DNA  [ **APEX1** ]  fragmentation showed that the TRX  [ **TXN** ]  gene suppressed hepatocyte apoptosis.  Ref: 12878175 Biochem Biophys Res Commun, 2003
- A covalent linkage between the gene 5 DNA  [ **APEX1** ]  polymerase of bacteriophage T7 and Eschericia italic coli italic Thioredoxin  [ **TXN** ] , the processivity factor Fate of Thioredoxin  [ **TXN** ]  during DNA  [ **APEX1** ]  synthesis.  Ref: 12692131 J Biol Chem< MedlineTA>J Biol Chem, 2003
- In contrast, we also found that the nuclear translocation of the redox regulators, Trx  [ **TXN** ]  and Ref 1  [ **APEX1** ] , increase with age, which was suppressed in CR rats.  Ref: 12742531 Exp Gerontol, 2003
- The TMX2 protein may possess an N terminal signal peptide, a potential transmembrane domain, an Myb DNA  [ **APEX1** ]  binding domain repeat signature, a thioredoxin  [ **TXN** ]  consensus pattern, an endoplasmic reticulum ER membrane retention signal KKXX like motif, and a dileucine motif in the tail.  Ref: 12670024 Biochem Genet, 2003
- The data from DNA  [ **APEX1** ]  microarray and Western blot analyses indicated that the levels of antiapoptotic proteins Bcl 2 and thioredoxin  [ **TXN** ]  2, which reside mainly in the mitochondria, are increased in RPMI8226 I cells.  Ref: 12467213 Mol Cancer Ther, 2002
